# Knowledge, attitudes, and practices among patients with combined dentition defect and non-functional impacted teeth toward tooth autotransplantation

**DOI:** 10.1186/s12903-024-04545-7

**Published:** 2024-07-04

**Authors:** Liang Zhao, Yuzhuan Hou, Juan Wang

**Affiliations:** Department of Stomatology, The First Affiliated Hospital of Xi’an Jiaotong University, Xi’an, 710000 China

**Keywords:** Knowledge, Attitudes, Practices, Cross-sectional study, Tooth autotransplantation

## Abstract

**Background:**

Tooth autotransplantation (TAT) is a surgical procedure involving the extraction of a tooth from one location and its subsequent transplantation into another alveolar socket within the same individual. This innovative treatment approach holds significant promise. Nonetheless, the potential recipients exhibit a limited level of awareness and understanding of this procedure. This study investigated the knowledge, attitudes, and practices (KAP) among patients with combined dentition defects and non-functional impacted teeth toward TAT.

**Methods:**

This web-based cross-sectional study was conducted between December 2022 and February 2023 at one hospital. A self-designed questionnaire was developed to collect demographic information of the patients and assess their knowledge, attitudes, and practices toward TAT.

**Results:**

A total of 533 valid questionnaires were collected. The mean knowledge, attitude, and practice scores were 5.55 ± 2.38 (possible range: 0–10), 26.82 ± 2.46 (possible range, 8–40), and 27.45 ± 7.40 (possible range, 9–45), respectively.

**Conclusion:**

The participants had insufficient knowledge, negative attitudes, and passive practices toward TAT. Targeted interventions should be implemented to improve the understanding and practice of TAT among patients with dentition defects.

**Supplementary Information:**

The online version contains supplementary material available at 10.1186/s12903-024-04545-7.

## Background

Dentition defects, including partial tooth loss, lead to abnormal tooth alignment [1]. Such defects can adversely impact patients’ pronunciation, masticatory function, aesthetic appearance, and overall health of their oral and maxillofacial systems [2]. Tooth autotransplantation (TAT) is a surgical procedure that involves extracting a tooth from one location and transplanting it into another alveolar socket within the same individual. This technique is commonly used for relocating impacted, unerupted, misplaced, or ectopically erupted teeth or in cases involving missing teeth requiring extraction [3, 4]. TAT offers several advantages, including facilitation of implant movement with adjacent teeth, promotion of bone regeneration in the surrounding area, good biocompatibility, cost-effectiveness, and short treatment duration [5–7]. Considering the benefits, TAT may serve as an effective strategy to overcome the limitations associated with traditional dental defect treatments. It is essential for the general population to be aware of this intervention’s potential.

Previous studies have evaluated the knowledge, attitudes, and practices (KAP) of TAT among residents specializing in oral and maxillofacial surgery (OMFS) and pediatric dentistry [8, 9]. These studies revealed that OMFS residents demonstrated insufficient knowledge concerning TAT. Since healthcare providers are a primary source of health-related information, it is crucial to incorporate TAT into the dental curriculum and standard OMFS textbooks to address this gap. In addition, practical training during the post-graduation period should be provided. Although most pediatric dentists are familiar with the TAT procedure, they have limited practical experience due to inadequate training. Moreover, several studies have identified that conducting KAP surveys can help identify gaps, misunderstandings, misconceptions, and deficiencies concerning autogenous dental implants within these three dimensions [10–12]. Nevertheless, no published studies or surveys assessed the KAP toward TAT among patients with combined dentition defects and non-functional impacted teeth.

Therefore, this study aimed to assess the KAP toward TAT in this population. The findings could contribute to developing educational interventions to enhance the general population’s KAP regarding TAT.

## Methods

### Study design and participants

This web-based cross-sectional study was conducted between December 1st, 2022, and February 18th, 2023, at one hospital among patients with combined dentition defects and non-functional impacted teeth. A self-designed questionnaire was developed to collect the demographic information of the patients and assess their KAP toward TAT. The study included participants who met the following criteria: (1) age between 18 and 45 years old, (2) presence of dentition defect requiring repair, and (3) presence of non-functional impacted teeth. Questionnaires containing blank items or logically flawed responses were excluded from this study. This study was ethically approved by the Ethics Committee of the First Affiliated Hospital of Xi’an Jiaotong University (ethical approval No. XJTU1AF2023LSK-326), and informed consent was obtained from the participants.

### Questionnaire introduction and collection

The questionnaire was developed with guidance from the Chinese Expert Consensus on Standardized Procedures for Tooth Autotransplantation [13] and the relevant literature [5, 14]. It was further modified based on feedback from three senior experts (Dr. Tao Hong, Chief Physician in Oral Implantology with 38 years of experience; Dr. Liu Xiuli, Associate Chief Physician in Prosthodontics with 40 years of experience; and Dr. Wang Xuerong, Associate Chief Physician in Oral and Maxillofacial Surgery with 42 years of experience.) and then pilot-tested on a small scale (*n* = 53), with a Cronbach’s α coefficient of 0.855, indicating good internal consistency.

The final questionnaire was in Chinese and contained four dimensions: demographic information, knowledge, attitudes, and practices. The demographic information consisted of 14 items, while the knowledge, attitudes, and practices comprised 11, eight, and nine items, respectively. The knowledge items were scored 1 point for correct answers and 0 points for incorrect answers. Two logically contradictory trap questions, namely “Dentition defects may have an effect on the individual’s pronunciation” (K3) and “The dentition defect does not have any effect on the individual’s pronunciation” (K10), were excluded from statistical analysis, resulting in a possible score range of 0 to 9. The attitude and practice items scored on a 5-point Likert scale ranging from very positive (5 points) to very negative (1 point). The possible score ranges were 8 to 40 for attitudes and 9 to 45 for practices. Data collection was performed using an online questionnaire hosted on Sojump (http://www.sojump.com).

The participants were recruited through convenience sampling when they presented to the hospital for a consultation. The online questionnaire was disseminated to the study participants through popular social media platforms such as WeChat. The participants simply had to log into the questionnaire, read and sign the informed consent form, and complete the questionnaire.

### Statistical analysis

The sample size was calculated using the formula

$$n=\left(\frac{{Z}_{1-\raisebox{1ex}{$\alpha $}\!\left/ \!\raisebox{-1ex}{$2$}\right.}}{\delta }\right)\times p\times (1-p)$$

where *Z*_*1−α/2*_ equals 1.96, *δ* is the allowable margin of error, usually set to 0.05, and *p* is set to 0.5 since the value is maximized when *p* = 0.5. Therefore, the sample size for the survey should be about 384 participants. Allowing for a questionnaire recovery rate of 80%, 480 questionnaires should be distributed.

STATA 17.0 (Stata Corporation, College Station, TX, USA) was used for statistical analysis. The continuous variables were expressed as means ± standard deviations. The categorical variables were expressed as n (%). The continuous variables that conformed to a normal distribution were tested using Student’s t-test or ANOVA. Participants who scored in the top 75% were deemed to possess sufficient knowledge, positive attitudes, and proactive practices. Multivariable logistic regression analysis was used to identify the risk factors for KAP. The cutoff scores for sufficient knowledge, active attitudes, and proactive practices were set at 8, 28, and 32, respectively. Two-sided *P* < 0.05 was considered statistically significant.

## Results

A total of 533 valid questionnaires were collected. Among the 533 participants, 249 (46.72%) were male, 380 (71.29%) resided in urban areas, and 435 (81.61%) possessed a junior college or bachelor’s degree. The causes of dentition defects included external traumas (7.88%), periodontal disease (17.45%), dental defects (10.13%), and caries (33.58%). The mean knowledge, attitudes, and practices scores were 5.55 ± 2.38 (possible range: 0–9), 26.82 ± 2.46 (possible range, 8–40), and 27.45 ± 7.40 (possible range, 9–45), respectively. The knowledge varied among participants with different monthly per capita income (*P* = 0.001), causes of dentition defects (*P* < 0.001), tooth loss locations (*P* < 0.001), frequency of brushing teeth per day (*P* = 0.008), condition of bleeding gums or sore teeth (*P* = 0.002), and alcohol consumption (*P* = 0.028). Similarly, the attitude scores differed among participants living in different residence areas (*P* = 0.001) and smoking habits (*P* = 0.003). The practice scores also showed variations among participants with different occupations (*P* = 0.001), causes of dentition defects (*P* < 0.001), tooth loss locations (*P* < 0.001), frequency of brushing teeth per day (*P* < 0.001), condition of bleeding gums or sore teeth (*P* = 0.002), and alcohol consumption (*P* = 0.019) (Table 1).

**Table 1:** Demographic characteristics and KAP score

Variables	*N* (%)	Knowledge score		Attitude score		Practice score	
		Mean ± SD	*P*	Mean ± SD	*P*	Mean ± SD	*P*
**Total score**	533	5.55 ± 2.38		26.82 ± 2.46		27.45 ± 7.40	
**Gender**			0.063		0.096		0.624
Male	249 (46.72)	5.34 ± 2.46		26.63 ± 2.48		27.62 ± 7.81	
Female	284 (53.28)	5.73 ± 2.30		26.99 ± 2.43		27.30 ± 7.03	
**Age**	26.51 ± 6.06		-				-
**Residence**			0.701		0.001		0.359
Rural	153 (28.71)	5.48 ± 2.39		26.24 ± 2.44		26.99 ± 8.06	
Urban	380 (71.29)	5.57 ± 2.37		27.05 ± 2.43		27.64 ± 7.12	
**Region**			0.104		0.422		0.737
Northern China	31 (5.82)	6.23 ± 1.84		27.00 ± 2.13		28.61 ± 7.22	
Eastern China	30 (5.63)	5.73 ± 2.30		26.77 ± 2.16		27.53 ± 6.80	
Northeast China	9 (1.69)	4.89 ± 2.26		28.22 ± 2.22		25.00 ± 5.98	
Southern China	32 (6.00)	4.88 ± 2.52		26.41 ± 2.11		26.38 ± 8.03	
Central China	15 (2.81)	6.40 ± 2.10		27.33 ± 3.06		26.40 ± 8.26	
Southwest China	19 (3.56)	6.37 ± 1.71		27.42 ± 2.43		29.00 ± 5.50	
Northwest China	397 (74.48)	5.48 ± 2.43		26.76 ± 2.51		27.46 ± 7.49	
**Marital status**			0.882		0.640		0.174
Unmarried/divorced/widowed	346 (64.92)	5.53 ± 2.43		26.78 ± 2.43		27.13 ± 7.42	
Married	187 (35.08)	5.57 ± 2.28		26.89 ± 2.52		28.04 ± 7.34	
**Education**			0.268		0.161		0.671
Middle school and below	6 (1.13)	4.00 ± 1.26		27.67 ± 2.25		28.00 ± 7.32	
High school and technical secondary school	22 (4.13)	5.59 ± 2.22		26.73 ± 2.12		28.59 ± 7.29	
Junior college and with a bachelor’s degree	435 (81.61)	5.51 ± 2.43		26.72 ± 2.52		27.52 ± 7.47	
Master’s degree or above	70 (13.13)	5.87 ± 2.09		27.39 ± 2.14		26.59 ± 7.09	
**Occupation**			0.144		0.065		0.001
Employed	176 (33.02)	5.42 ± 2.38		27.09 ± 2.58		26.68 ± 7.32	
Freelancer	89 (16.70)	5.30 ± 2.44		26.42 ± 2.44		30.38 ± 7.29	
Student	229 (42.96)	5.81 ± 2.28		26.68 ± 2.29		26.85 ± 7.47	
Unemployed	39 (7.32)	5.13 ± 2.67		27.36 ± 2.72		27.77 ± 7.09	
**Monthly per capita income (CNY)**			0.001		0.445		0.128
<2000	50 (9.38)	5.72 ± 2.52		26.24 ± 2.83		27.88 ± 7.36	
2000–5000	145 (27.20)	4.97 ± 2.63		26.93 ± 2.49		27.36 ± 7.45	
5000-10,000	177 (33.21)	5.63 ± 2.18		26.95 ± 2.19		28.37 ± 6.98	
10,000–20,000	109 (20.45)	6.25 ± 2.10		26.79 ± 2.68		26.78 ± 7.42	
>20,000	52 (9.76)	5.25 ± 2.34		26.69 ± 2.37		25.58 ± 8.38	
**Causes of dentition defect**			< 0.001		0.115		<0.001
External traumas	42 (7.88)	5.12 ± 2.23		26.83 ± 3.49		30.52 ± 7.03	
Periodontal disease	93 (17.45)	5.65 ± 2.39		26.33 ± 2.11		29.84 ± 5.87	
Dental defects	54 (10.13)	4.96 ± 2.31		27.39 ± 2.67		26.22 ± 7.57	
Caries	179 (33.58)	6.24 ± 1.99		26.97 ± 2.44		28.48 ± 6.67	
Other	165 (30.96)	5.04 ± 2.63		26.74 ± 2.24		24.61 ± 7.98	
**Tooth Loss Locations**			< 0.001		0.137		<0.001
1–2 loss locations	483 (90.62)	5.40 ± 2.37		26.84 ± 2.49		27.05 ± 7.40	
3–4 loss locations	44 (8.26)	7.16 ± 1.52		26.89 ± 2.00		31.80 ± 5.53	
5–6 loss locations	6 (1.13)	5.67 ± 3.67		24.68 ± 2.32		27.83 ± 10.32	
**Frequency of Brushing Teeth per Day**			0.008		0.124		<0.001
1 time	60 (11.26)	5.00 ± 2.58		26.93 ± 2.10		23.72 ± 7.01	
2 times	395 (74.11)	5.73 ± 2.31		26.91 ± 2.41		27.76 ± 7.16	
3 times and above	78 (14.63)	5.01 ± 2.44		26.29 ± 2.88		28.77 ± 8.07	
**Symptoms such as bleeding gums or sore teeth**			0.002		0.896		0.002
Yes	298 (55.91)	5.83 ± 2.32		26.83 ± 2.40		28.35 ± 7.04	
No	235 (44.09)	5.19 ± 2.40		26.80 ± 2.53		26.31 ± 7.70	
**Smoking behavior**			0.089		0.003		0.345
Yes	148 (27.77)	5.26 ± 2.45		26.30 ± 2.57		27.94 ± 8.08	
No	385 (72.23)	5.65 ± 2.34		27.02 ± 2.39		27.26 ± 7.12	
**Alcohol consumption**			0.028		0.082		0.019
Yes	188 (35.27)	5.24 ± 2.51		26.57 ± 2.71		28.47 ± 8.06	
No	345 (64.73)	5.71 ± 2.29		26.96 ± 2.30		26.90 ± 6.96	

In the knowledge dimension, the question that exhibited the highest accuracy rate (89.68%) was about the impact of dentition defects on an individual’s pronunciation (K3). Conversely, the question with the lowest accuracy rate (45.78%) revolved around TAT, which encompasses the surgical relocation of a tooth within the same individual. Remarkably, this procedure allows the use of any tooth, such as a transplanted one, and lacks specific indications (K5). Furthermore, the correctness rate for the statement “Tooth autotransplantation can be performed even if the patient has a severe metabolic bone disease” (K11) was low, at 51.97% (Table 2).

**Table 2:** Knowledge

Knowledge	Correctness, *N* (%)
K1. Dentition defects refer to the incomplete development of permanent dentition caused by the partial absence of teeth, resulting in diminished chewing efficiency and a lower quality of life.	423 (79.36)
K2. Dentition defects can not only lead to the misalignment of neighboring teeth but also have a detrimental impact on the digestive system and the psychological well-being of the affected individual.	406 (76.17)
K3. Dentition defects can affect an individual’s ability to pronounce sounds accurately.	478 (89.68)
K4. The treatment options for dentition defects generally involve partial dentures, fixed dental prostheses, dental implants, and tooth autotransplantation.	394 (73.92)
K5. Tooth autotransplantation is a surgical procedure in which a tooth is transplanted from one location to another within the same individual. It is possible to use any tooth for this procedure, and there are no specific indications or restrictions.	244 (45.78)
K6. Tooth autotransplantation is not recommended if the individual presents with severe oral or underlying diseases, such as advanced periodontitis or metabolic bone disorders.	347 (65.10)
K7. The advantage of tooth autotransplantation is that, upon successful healing, it closely mimics the natural tissue structure, resembling a normal tooth.	390 (73.17)
K8. The drawback of tooth autotransplantation is the uncertainty regarding its long-term prognosis, which may necessitate subsequent root canal treatment or full crown restoration.	336 (63.04)
K9. Patients who undergo tooth autotransplantation should diligently monitor their oral and overall health conditions, such as the risk of infection, and adhere to medical advice for regular follow-up.	416 (78.05)
K10. Dentition defects do not affect an individual’s ability to pronounce sounds accurately.	478 (89.68)
K11. Tooth autotransplantation can still be considered as a treatment option even if the patient has severe metabolic bone disease.	277 (51.97)

Regarding attitudes, over 80% of respondents strongly agree or agree that acquiring knowledge about dental abnormalities and their corresponding treatments is important (A1). About 87% of them also agree in terms of adhering to doctor’s instructions, timely medication intake, and maintaining attention towards prognosis (A3) and the idea of communicating with their doctors regarding their conditions (A2). Approximately 79% of them exhibited confidence in the treatment options selected by their doctors (A6). Moreover, the responses for A4, A5, and A7 indicated that over 70% of the respondents recognize the benefits of employing TAT in MRI and CT examinations (A4), albeit expressing concerns about the possibility of post-operative teeth loosening (A5) and procedure failure (A7). Approximately 67% of the respondents strongly agree or agree that the decision to choose TAT is primarily influenced by economic considerations (A8) (Table 3).

**Table 3:** Attitudes

Attitude	Strongly agree	Agree	Uncertain	Disagree	Strongly disagree
A1. It is essential for patients to pay attention and acquire knowledge about dental abnormalities, such as dentition defects, and the corresponding treatments, including tooth autotransplantation.	171(32.08)	258(48.41)	97(18.20)	5(0.94)	2(0.38)
A2. You are highly willing to engage in discussions with your attending physician regarding your condition.	214(40.15)	252(47.28)	50(9.38)	13(2.44)	4(0.75)
A3. It is crucial to adhere to your doctor’s instructions, adhere to medication schedules, and remain attentive to your own prognosis throughout the course of treatment.	233(43.71)	233(43.71)	62(11.63)	4(0.75)	1(0.19)
A4. You believe that the presence of metallic implants in the body following dental implantation may impede future MRI and CT examinations. In contrast, tooth autotransplantation does not cause such interference, thus acknowledging the advantages of this procedure.	158(29.64)	231(43.34)	127(23.83)	15(2.81)	2(0.38)
A5. You may find it challenging to tolerate any loosening of the teeth following the surgery.	160(30.02)	263(49.34)	88(16.51)	19(3.56)	3(0.56)
A6. You have complete confidence in the treatment options selected by your doctor.	152(28.52)	275(51.59)	90(16.89)	12(2.25)	4(0.75)
A7. You harbor apprehension regarding the potential failure of the procedure.	140(26.27)	269(50.47)	98(18.39)	18(3.38)	8(1.50)
A8. Your choice of tooth autotransplantation is more of an economic consideration, and you may prefer a treatment option other than tooth autotransplantation if you are in a better financial state.	126(23.64)	234(43.90)	143(26.83)	28(5.25)	2(0.38)

The results from the practices revealed that more than 40% of the respondents expressed a higher level of concern regarding metabolic-related bone diseases (P6), and over 60% were willing to recommend TAT to others facing the same condition as themselves (P9). Additionally, over 70% of the participants maintained good oral health and practiced proper oral hygiene routines 3–4 or 5–6 times per week in their daily lives (P7). Moreover, more than 60% of the respondents were open to considering TAT as a viable treatment option (P8). However, only approximately 30% of the participants consistently or frequently visited a dental clinic or hospital dentist for regular dental checkups (P1), received regular professional dental care (P2), actively sought knowledge about dental abnormalities and TAT (P3), and expressed concern about tooth misalignment (P4) or periodontal disease (P5) (Table 4).

**Table 4:** Practices

Statements	Always	Often	Sometimes	Occasionally	Never
P1. How often do you visit a dental clinic or hospital dentist for regular dental checkups?	78 (14.63)	16 (21.76)	101 (21.76)	179 (33.58)	59 (11.07)
P2. How often do you have regular professional dental care (e.g., deep oral cleaning, dental scaling, etc.)?	56 (10.51)	102 (19.14)	105 (19.70)	189 (35.46)	81 (15.20)
P3. How often do you proactively acquire knowledge about dentition defects and tooth autotransplantation through various sources (e.g., in-hospital education, via TV and the internet, or by consulting your attending doctor, etc.)?	62 (11.63)	96 (18.01)	118 (22.14)	161 (30.21)	96 (18.01)
P4. How often are you concerned about the presence or absence of tooth deformity in yourself?	83 (15.57)	123 (23.08)	131 (24.58)	132 (24.77)	64 (12.01)
P5. How often are you concerned about the presence or absence of periodontitis in yourself?	72 (13.51)	100 (18.76)	149 (27.95)	128 (24.02)	84 (15.76)
	**Very conforming**	**Conforming**	**Uncertain**	**Not conforming**	**Very non-conforming**
P6. You are more concerned about the presence or absence of metabolic-related bone disease in yourself.	70 (13.13)	164 (30.77)	144 (27.02)	102 (19.14)	53 (9.94)
P9. You would recommend tooth autotransplantation to a patient in the same condition as you.	107 (20.08)	216 (40.53)	170 (31.89)	27 (5.07)	13 (2.44)
	**1 ~ 2 times a week**	**3 ~ 4 times a week**	**5 ~ 6 times a week**	**Every day**	**Hardly never**
P7. How often do you maintain good oral health and good oral hygiene practices (e.g., use of mouthwash, flossing to clean up food debris, etc.) in your daily life?	27 (5.07)	170 (31.89)	216 (40.53)	107 (20.08)	13 (2.44)
	**Very willing**	**Willing**	**Refuse**	**Firmly Refuse**	**Didn’t think about it**
P8. You are willing to choose tooth autotransplantation as an option for treatment	96 (18.01)	257 (48.22)	34 (6.38)	6 (1.13)	140 (26.27)

The multivariable logistic regression analysis showed that periodontal disease (OR = 3.01, 95%CI: 1.04–8.71, *P* = 0.042), caries (OR = 3.71, 95%CI: 1.36–10.16, *P* = 0.011), 3–4 tooth loss locations (OR = 5.29, 95%CI: 2.66–10.55, *P* < 0.001), and bleeding gums or sore teeth history (OR = 1.86, 95%CI: 1.21–2.88, *P* < 0.001) were independently associated with sufficient knowledge (Table S1). Urban residence (OR = 2.19, 95% CI: 1.45–3.31, *P* < 0.001) was independently associated with positive attitudes (Table S1) Attitude (OR = 1.11, 95% CI: 1.03–1.21, *P* = 0.009), three or more times of Brushing Teeth per Day (OR = 3.49, 95% CI: 1.52–7.99, *P* = 0.003), alcohol consumption (OR = 2.31, 95% CI: 1.39–3.83, *P* = 0.001), dental defects (OR = 0.40, 95% CI: 0.16–0.98, *P* = 0.044), and other causes of dentition defect (OR = 0.32, 95% CI: 0.15–0.67, *P* = 0.003) were independently associated with proactive practices (Table S1).

## Discussion

The participants had insufficient knowledge, negative attitudes, and passive practices toward TAT. Targeted interventions should be implemented to improve the understanding and practice of TAT among patients with dentition defects.

Previous studies have explored the knowledge, attitudes, and practices toward TAT among dentists and have reported inadequate understanding and practice [8, 9, 15]. However, there is a lack of studies focusing on patients.

In this study, it was found that patients had insufficient knowledge of TAT. In order to bridge this knowledge gap, targeted education should be provided to enhance patients’ understanding of TAT indications [3, 6, 16]. Moreover, the multivariable analysis revealed that participants with dental defects caused by periodontal disease and caries exhibited better knowledge compared to those with defects resulting from external trauma. This discrepancy may be attributed to the regular hospital visits of patients with periodontal disease and caries, which could motivate them to learn about dentistry, including TAT and dental defects [17]. In addition, a history of bleeding gums or sore teeth was another factor influencing the knowledge scores. As bleeding gums or sore teeth are common symptoms of dental diseases, individuals who have experienced these symptoms may have greater exposure to dental knowledge [18, 19].

Regarding attitudes, a considerable portion of the participants expressed concerns about surgical failure and complications despite preferring the benefits of TAT in MRI and CT examinations. Thus, dentists must meticulously assess the indications for a given procedure, considering patient selection and the condition of immature teeth [20]. Fortunately, most participants expressed strong agreement or agreement regarding the significance of acquiring knowledge about dental anomalies and their corresponding treatments. They emphasized the importance of diligently adhering to doctors’ instructions, ensuring timely medication intake, and displaying attentiveness toward prognosis. Moreover, many participants demonstrated a willingness to communicate openly with their healthcare providers regarding their specific conditions, exhibiting a sense of trust in the treatment options recommended by the doctors.

In terms of practices, the average scores for practices were substandard, indicating insufficient adherence to proper oral health habits by the participants. Only about 30% of the participants frequently visited dental clinics or hospital dentists for regular dental checkups, received regular professional dental care, proactively sought knowledge about dentition defects and TAT, and expressed concerns about tooth deformity or periodontitis. Several studies have also demonstrated suboptimal oral health management practices among various populations [21, 22]. Although many people prioritize maintaining oral health habits, only a small percentage undergo regular dental checkups and receive dental care [23]. Educational interventions conducted by healthcare professionals in their practice settings have the potential to address this issue [24–26]. More than 60% of the participants in this study expressed willingness to choose TAT as a treatment option and recommend it to others with similar conditions, indicating a positive acceptance of the procedure. It may be attributed to the fact that TAT uses natural teeth to restore missing teeth, aligning with patients’ treatment expectations [27].

This study retains significant clinical relevance as it highlights several issues about TAT, including inadequate knowledge, negative attitudes, and passive engagement. These findings serve as a crucial reminder to healthcare professionals about the importance of customizing interventions based on patient’s requirements. Healthcare providers can enhance patients’ comprehension of the subject by delivering targeted education and information about TAT, fostering positive attitudes, and elevating their practical proficiency.

This study has several limitations. As a cross-sectional study, it is unable to establish causal relationships. The results rely on self-reported questionnaires, which may be influenced by recall bias and a desire to present their clinical knowledge and experience positively. The participants were from a single hospital and a limited geographical area, limiting generalizability.

## Conclusion

In conclusion, the participants had insufficient knowledge, negative attitudes, and passive practices toward TAT. Targeted interventions should be implemented to improve the understanding and practice of TAT among patients with dentition defects.

### Electronic supplementary material

Below is the link to the electronic supplementary material.

Supplementary Material 1

## Data Availability

All data generated or analyzed during this study are included in this article.

## Abbreviations

TAT: Tooth autotransplantation
KAP: Knowledge, attitudes, and practices
OMFS: Oral and maxillofacial surgery
