# Supplementary material for: Knowledge, attitudes, and practices among patients with combined dentition defect and non-functional impacted teeth toward tooth autotransplantation
Source: BMC Oral Health. 2024 Jul 4;24:761. doi: 10.1186/s12903-024-04545-7 (PMC11225189; doi:10.1186/s12903-024-04545-7)
Supplement: Supplementary file 1 — Supplementary Material 1 [file 12903_2024_4545_MOESM1_ESM.docx]

Table **S1**. Multivariate logistic regression analysis of knowledge, attitudes and practices

|  | **Univariable** | | **Multivariable** | |
| --- | --- | --- | --- | --- |
|  | **OR (95%CI)** | **P value** | **OR (95%CI)** | **P value** |
| **Knowledge** |  |  |  |  |
| **Gender** |  |  |  |  |
| Male | Ref. |  |  |  |
| Female | 1.07 (0.73 1.47) | 0.724 |  |  |
| **Age** | 1.00 (0.97 1.03) | 0.888 |  |  |
| **Residence** |  |  |  |  |
| Rural | Ref. |  |  |  |
| Urban | 1.16 (0.75 1.78) | 0.510 |  |  |
| **Marital status** |  |  |  |  |
| Unmarried/divorced/widowed | Ref. |  |  |  |
| Married | 1.33 (0.89 1.97) | 0.162 |  |  |
| **Occupations** |  |  |  |  |
| Employed | 1.18 (0.50 2.76) | 0.708 |  |  |
| Freelancer | 1.60 (0.65 3.94) | 0.307 |  |  |
| Student | 1.64 (0.72 3.74) | 0.243 |  |  |
| Unemployed | Ref. |  |  |  |
| **Monthly per capita income (CNY)** |  |  |  |  |
| <2000 | Ref. |  |  |  |
| 2000-5000 | 0.68 (0.32 1.45) | 0.319 |  |  |
| 5000-10,000 | 1.12 (0.55 2.28) | 0.754 |  |  |
| 10,000-20,000 | 1.78 (0.85 3.74) | 0.125 |  |  |
| >20,000 | 0.68 (0.27 1.73) | 0.415 |  |  |
| **Causes of dentition defect** |  |  |  |  |
| External traumas | Ref. |  | Ref. |  |
| Periodontal disease | 4.26 (1.53 11.88) | 0.006 | 3.01 (1.04 8.71) | 0.042 |
| Dental defects | 1.10 (0.32 3.75) | 0.876 | 0.98 (0.28 3.45) | 0.975 |
| Caries | 4.02 (1.50 10.74) | 0.006 | 3.71 (1.36 10.16) | 0.011 |
| Other | 1.92 (0.70 5.26) | 0.204 | 2.17 (0.78 6.09) | 0.140 |
| **Tooth Loss Locations** |  |  |  |  |
| 1-2 loss locations | Ref. |  | Ref. |  |
| 3-4 loss locations | 6.56 (3.39 12.67) | <0.001 | 5.29 (2.66 10.55) | <0.001 |
| 5-6 loss locations | 6.78 (1.23 37.52) | 0.028 | 5.38 (0.93 31.27) | 0.061 |
| **Frequency of Brushing Teeth per Day** |  |  |  |  |
| 1 time | Ref. |  |  |  |
| 2 times | 1.81 (0.91 3.60) | 0.092 |  |  |
| 3 times and above | 1.34 (0.58 3.09) | 0.499 |  |  |
| **Symptoms such as bleeding gums or sore teeth** |  |  |  |  |
| Yes | 2.16 (1.44 3.24) | <0.001 | 1.86 (1.21 2.88) | <0.001 |
| No | Ref. |  | Ref. |  |
| **Smoking behavior** |  |  |  |  |
| Yes | 1.11 (0.73 1.70) | 0.617 |  |  |
| No | Ref. |  |  |  |
| **Alcohol consumption** |  |  |  |  |
| Yes | 0.98 (0.66 1.47) | 0.928 |  |  |
| No | Ref. |  |  |  |
| **Attitudes** |  |  |  |  |
| **Knowledge** | 1.08 (1.00 1.16) | 0.050 | 1.08 (1.00 1.16) | 0.054 |
| **Gender** |  |  |  |  |
| Male | Ref. |  |  |  |
| Female | 1.22 (0.86 1.73) | 0.266 |  |  |
| **Age** | 1.00 (0.97 1.03) |  |  |  |
| **Residence** |  |  |  |  |
| Rural | Ref. |  | Ref. |  |
| Urban | 2.19 (1.45 3.31) | <0.001 | 2.19 (1.45 3.31) | <0.001 |
| **Marital status** |  |  |  |  |
| Unmarried/divorced/widowed | Ref. |  |  |  |
| Married | 0.99 (0.69 1.43) | 0.959 |  |  |
| **Occupation** |  |  |  |  |
| Employed | 0.87 (0.43 1.74) | 0.687 |  |  |
| Freelancer | 0.62 (0.29 1.34) | 0.227 |  |  |
| Student | 0.65 (0.33 1.29) | 0.219 |  |  |
| Unemployed | Ref. |  |  |  |
| **Monthly per capita income (CNY)** |  |  |  |  |
| <2000 | Ref. |  |  |  |
| 2000-5000 | 1.09 (0.56 2.11) | 0.803 |  |  |
| 5000-10,000 | 1.09 (0.57 2.08) | 0.787 |  |  |
| 10,000-20,000 | 0.91 (0.45 1.82) | 0.787 |  |  |
| >20,000 | 0.94 (0.42 2.10) | 0.879 |  |  |
| **Causes of dentition defect** |  |  |  |  |
| External traumas | Ref. |  |  |  |
| Periodontal disease | 0.60 (0.28 1.28) | 0.189 |  |  |
| Dental defects | 1.33 (0.59 3.00) | 0.487 |  |  |
| Caries | 0.88 (0.44 1.730 | 0.704 |  |  |
| Other | 0.78 (0.39 1.56) | 0.484 |  |  |
| **Tooth Loss Locations** |  |  |  |  |
| 1-2 loss locations | Ref. |  |  |  |
| 3-4 loss locations | 0.98 (0.52 1.85) | 0.949 |  |  |
| 5-6 loss locations | -- | -- |  |  |
| **Frequency of Brushing Teeth per Day** |  |  |  |  |
| 1 time | Ref. |  |  |  |
| 2 times | 0.84 (0.49 1.46) | 0.546 |  |  |
| 3 times and above | 0.62 (0.31 1.24) | 0.175 |  |  |
| **Symptoms such as bleeding gums or sore teeth** |  |  |  |  |
| Yes | 0.90 (0.64 1.28) | 0.570 |  |  |
| No | Ref. |  |  |  |
| **Smoking behavior** |  |  |  |  |
| Yes | 0.75 (0.50 1.11) | 0.153 |  |  |
| No | Ref. |  |  |  |
| **Alcohol consumption** |  |  |  |  |
| Yes | 0.88 (0.61 1.27) | 0.496 |  |  |
| No | Ref. |  |  |  |
| **Practices** |  |  |  |  |
| **Knowledge** | 1.09 (1.01 1.18) | 0.033 | 1.06 (0.97 1.15) | 0.236 |
| **Attitude** | 1.08 (1.00 1.16) | 0.043 | 1.11 (1.03 1.21) | 0.009 |
| **Gender** |  |  |  |  |
| Male | Ref. |  | Ref. |  |
| Female | 0.69 (0.48 0.99) | 0.045 | 0.92 (0.58 1.07) | 0.733 |
| **Age** | 1.04 (1.01 1.07) | 0.016 | 1.02 (0.98 1.07) | 0.327 |
| **Residence** |  |  |  |  |
| Rural | Ref. |  |  |  |
| Urban | 0.86 (0.58 1.27) | 0.448 |  |  |
| **Marital status** |  |  |  |  |
| Unmarried/divorced/widowed | Ref. |  | Ref. |  |
| Married | 1.61 (1.11 2.33) | 0.012 | 0.94 (0.51 1.72) | 0.833 |
| **Occupations** |  |  |  |  |
| Employed | 0.69 (0.34 1.42) | 0.312 |  |  |
| Freelancer | 1.87 (0.87 4.04) | 0.109 |  |  |
| Student | 0.65 (0.32 1.31) | 0.228 |  |  |
| Unemployed | Ref. |  |  |  |
| **Monthly per capita income (CNY)** |  |  |  |  |
| <2000 | Ref. |  |  |  |
| 2000-5000 | 0.99 (0.50 1.97) | 0.971 |  |  |
| 5000-10,000 | 1.39 (0.71 2.71) | 0.332 |  |  |
| 10,000-20,000 | 1.09 (0.53 2.23) | 0.809 |  |  |
| >20,000 | 0.71 (0.30 1.68) | 0.434 |  |  |
| **Causes of dentition defect** |  |  |  |  |
| External traumas | Ref. |  | Ref. |  |
| Periodontal disease | 1.12 (0.54 2.33) | 0.754 | 1.03 (0.47 2.30) | 0.935 |
| Dental defects | 0.46 (0.20 1.07) | 0.073 | 0.40 (0.16 0.98) | 0.044 |
| Caries | 0.60 (0.30 1.18) | 0.137 | 0.63 (0.30 1.32) | 0.221 |
| Other | 0.31 (0.15 0.62) | 0.001 | 0.32 (0.15 0.67) | 0.003 |
| **Tooth Loss Locations** |  |  |  |  |
| 1-2 loss locations | Ref. |  | Ref. |  |
| 3-4 loss locations | 2.81 (1.50 5.26) | 0.001 | 1.95 (0.97 3.91) | 0.062 |
| 5-6 loss locations | 2.14 (0.43 10.71) | 0.356 | 1.55 (0.25 9.47) | 0.636 |
| **Frequency of Brushing Teeth per Day** |  |  |  |  |
| 1 time | Ref. |  | Ref. |  |
| 2 times | 1.84 (0.96 3.51) | 0.067 | 1.54 (0.76 3.10) | 0.231 |
| 3 times and above | 3.10 (1.45 6.62) | 0.003 | 3.49 (1.52 7.99) | 0.003 |
| **Symptoms such as bleeding gums or sore teeth** |  |  |  |  |
| Yes | 1.52 (1.06 2.19) | 0.025 | 1.24 (0.82 1.89) | 0.314 |
| No | Ref. |  | Ref. |  |
| **Smoking behavior** |  |  |  |  |
| Yes | 1.53 (1.03 2.26) | 0.033 | 0.92 (0.54 1.55) | 0.756 |
| No | Ref. |  | Ref. |  |
| **Alcohol consumption** |  |  |  |  |
| Yes | 2.04 (1.41 2.95) | <0.001 | 2.31 (1.39 3.83) | 0.001 |
| No | Ref. |  | Ref. |  |
